# Electrical antimicrobial susceptibility testing based on aptamer-functionalized capacitance sensor array for clinical isolates

**DOI:** 10.1038/s41598-020-70459-3

**Published:** 2020-08-13

**Authors:** Kyo-Seok Lee, Sun-Mi Lee, Jeseung Oh, In Ho Park, Jun Ho Song, Myeonggil Han, Dongeun Yong, Kook Jin Lim, Jeon-Soo Shin, Kyung-Hwa Yoo

**Affiliations:** 1 Department of Physics, Yonsei University, Seoul, 03722 Republic of Korea; 2 Nanomedical Graduate Program, Yonsei University, Seoul, 03722 Republic of Korea; 3 Proteomtech Inc., 1101 Wooree-Venture Town, Seoul, 07573 Republic of Korea; 4 Department of Microbiology, College of Medicine, Yonsei University, Seoul, 03722 Republic of Korea; 5 Severance Biomedical Science Institute and Institute for Immunology and Immunological Diseases, Yonsei University College of Medicine, Seoul, 03722 Republic of Korea; 6 Department of Laboratory Medicine and Research Institute of Bacterial Resistance, Yonsei University College of Medicine, Seoul, 03722 Republic of Korea

**Keywords:** Biological techniques, Biotechnology

## Abstract

To prescribe effective antibiotics to patients with bacterial infections in a timely manner and to avoid the misuse of antibiotics, a rapid antimicrobial susceptibility test (AST) is essential. However, conventional AST methods require more than 16 h to provide results; thus, we developed an electrical AST (*e*-AST) system, which provides results within 6 h. The proposed *e*-AST is based on an array of 60 aptamer-functionalized capacitance sensors that are comparable to currently available AST panels and a pattern-matching algorithm. The performance of the *e*-AST was evaluated in comparison with that of broth microdilution as the reference test for clinical strains isolated from septic patients. A total of 4,554 tests using *e*-AST showed a categorical agreement of 97% with a minor error of 2.2%, major error of 0.38%, and very major error of 0.38%. We expect that the proposed *e*-AST could potentially aid antimicrobial stewardship efforts and lead to improved patient outcomes.

## Introduction

Bacterial infection-related complications such as sepsis are serious diseases with mortality rates ranging from 30 to 50%^1–3^. If appropriate treatment is delayed, survival chances decrease drastically. Thus, rapid antimicrobial susceptibility testing (AST) is required to prescribe the patient with effective antibiotics and avoid their misuse^4^. However, conventional phenotype-based AST methods, including broth dilution and disk diffusion methods, require more than 3 d to show AST results^5,6^. Genotypic methods, such as PCR, enable the rapid identification of strains carrying specific antibiotic resistance genes^7^. However, they have several disadvantages, such as reliance on high bacterial concentrations to provide sufficient DNA and manual sample handling steps, such as lysing bacterial cells to extract nucleic acids. Further, the extracted DNA contains DNA from both living and dead cells, leading to a high false positive rate. Hence, phenotype methods remain the “gold standard” for clinical AST.

To reduce AST time, various phenotype AST techniques based on optical imaging^8–14^, microfluidic channels^15–19^, and other biosensors^4,20^ have been pursued. For example, several microscopy-based methods have been developed to measure bacterial growth every 10 min as clonal aggregates multiply in Mueller–Hinton media, to determine antibiotic-induced morphological changes in single bacterial cells^10^, and to measure the number of bacteria in a microfluidic channel^19^. In addition, rapid AST using biochemical, optical, and isothermal measurements has been reported^21,22^.

**Figure 1:**
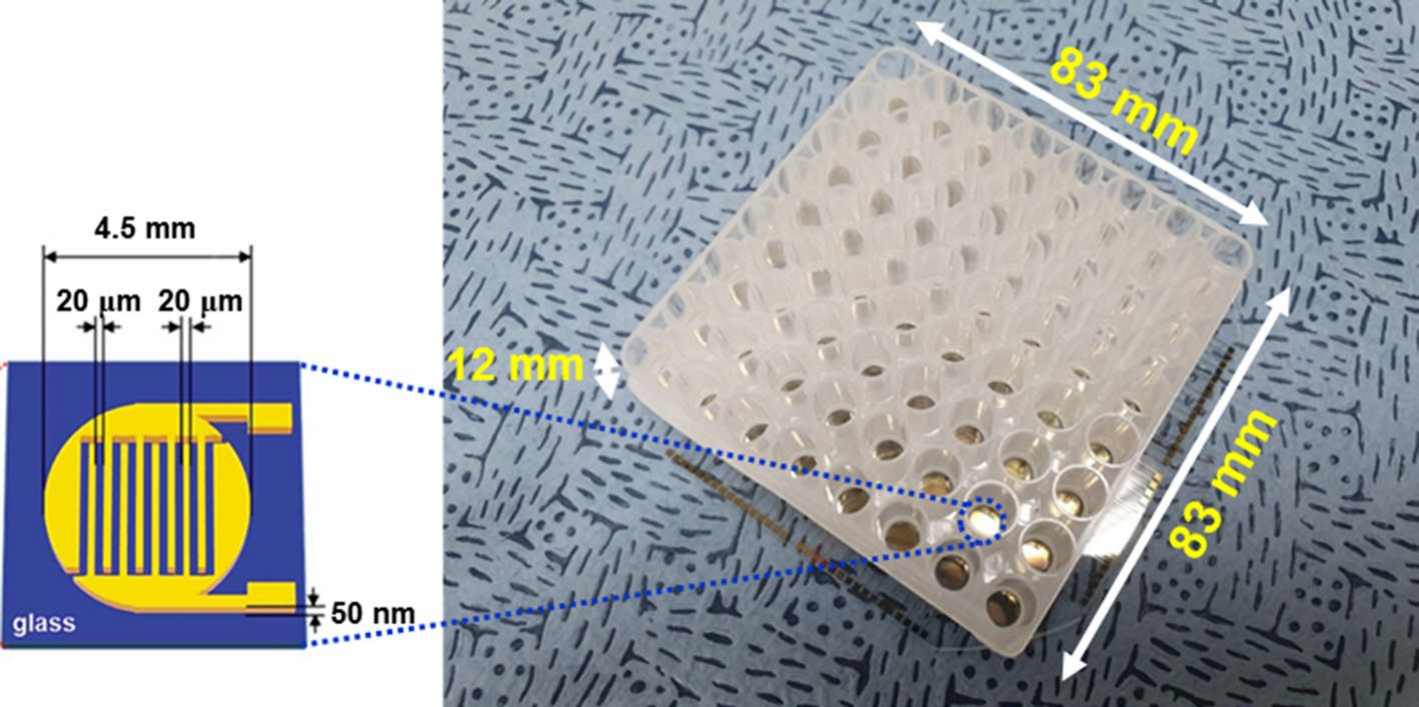
Photograph of capacitance sensor array consisting of 60 capacitance sensors. The inset shows a schematic of capacitance sensor.

Here, we report electrical AST (*e*-AST) chips composed of 60 aptamer-functionalized capacitance sensors (Fig. 1). Bacteria act as dielectric particles; thus, if bacteria are bound to the sensor surface via the aptamer, the capacitance changes. As a result, bacterial activities, such as growth and death, can be monitored in real-time by measuring the capacitance change, allowing more rapid AST^23,24^. For AST of clinical samples, it is necessary to simultaneously test various antimicrobial agents at different concentrations; therefore, we fabricated sensor arrays consisting of 60 sensors. Among the 60 sensors, two and three sensors were used for negative and positive controls, respectively, and the other 55 sensors were used to measure the antibiotic susceptibility to 11 antibiotics at five different concentrations (see Supplementary Table S1). To evaluate the performance of the *e*-AST system, 30 clinical strains, most of which are frequently found in sepsis, were tested (Table 1). The categorical agreement between the *e*-AST system and gold standard broth microdilution (BMD) test was estimated to be 97%, meeting the requirements of the United States Food and Drug Administration (FDA).

**Table 1:** Representative microorganism from sepsis in clinical strains.

Bacteria	Number of strains	Clinical strains
**Gram-negative**		
*E. coli*	5	U433, U556^a^, B12327, U5307, U6267^a^
*A. baumannii*	5	P762, T800, R4197, R4299, R4356
*P. aeruginoa*	5	PAE1, PAE2, PAE3, PAE4, PAE5
*K. pneumoniae*	5	KP1^a^, KP2^a^, KP3, KP4^a^, KP5^a^
**Gram-positive**		
*S. aureus*	5	P101, T82, C970, R4308, R4319
*E. faecalis*	5	R1238, U554, U5179, U4879, U5064

^a^Extended-spectrum β-lactamase (ESBL) positive strains.

## Materials and methods

### Fabrication of *e*-AST chip

The *e*-AST chip, consisting of 60 sensors, was fabricated on a glass substrate (Fig. 1). Each sensor had interdigitated Au electrodes with a width of 20 µm and spacing of 20 µm, which were patterned by conventional photolithography and lift-off techniques. For bacterial culture, a 300 μl-acrylic well (Nobel Biosciences Inc., Hwaseong-Si, South Korea) was attached to the array sensor using 3 M VHBTM tape. Next, the bacteria-aptamers were immobilized on the sensor surface.

### Bacterial strains and their growth condition

Thirty clinical isolates comprising four Gram-negative strains (*Escherichia coli*, *Acinetobacter baumannii*, *Klebsiella pneumoniae*, and *Pseudomonas aeruginosa*) and two Gram-positive strains (*Staphylococcus aureus* and *Enterococcus faecalis*) were used for the study (Table 1). Two extended-spectrum β-lactamase (ESBL)-producing *E. coli* and four ESBL-producing *K. pneumoniae* were included. Sepsis-causing clinical bacterial strains from various specimens (blood, pus, and urine) were collected from the laboratories of Yonsei University Severance Hospital (Seoul, South Korea). They were subcultured on blood agar plates (KOMED Life Science Co., Ltd., Seoul, South Korea) for 20 to 24 h before the test and their antimicrobial resistance was performed with VITEK 2 systems (bioMerieux with three AST cards). Mueller Hinton Broth (cation-adjusted) (BD Biosciences, San Jose, CA, USA) was used as the growth medium. This study was approved by the Institutional Review Board of Severance Hospital, Yonsei University Health System (4-2017-1179).

### Aptamer immobilization on the sensor surface

The sequences of the DNA aptamers, specific to each bacterial species, were as follows: *E. coli*, 5′-GCA ATG GTA CGG TAC TTC CCC ATG AGT GTT GTG AAA TGT TGG GAC ACT AGG TGG CAT AGA GCC GCA AAA GTG CAC GCT ACT TTG CTA A-3′^25^; *A. baumannii*, 5′-TAC ATG GTC AAC CAA ATT CTT GCA AAT TCT GCA TTC CTA CTG T-3′^26^; *K. pneumoniae*, 5′-GCA ATG GTA CGG TAC TTC C(N45)-CAA AAG TGC ACG CTA CTT TGC TAA-3′^27^; *P. aeruginosa*, 5′-CCC CCG TTG CTT TCG CTT TTC CTT TCG CTT TTG TTC GTT TCG TCC CTG CTT CCT TTC TTG-(CH_2_)_3_′-SH-3′^28^; *S. aureus*, 5′-GCA ATG GTA CGG TAC TTC CTC GGC ACG TTC TCA GTA GCG CTC GCT GGT CAT CCC ACA GCT ACG TCA AAA GTG CAC GCT ACT TTG CTA A-3′^29^; and *E. faecalis*, 5′-ATC CAG AGT GAC GCA GCA CGA CAC GTT AGG TTG GTT AGG TTG GTT AGT TTC TTG TGG ACA CGG TGG CTT A-3′^30^. All aptamers were custom synthesized by Genotech Inc. (Daejeon, South Korea) and dissolved at 10 μM in distilled water for further use.

To detect various bacteria, mixtures of various bacterial aptamers were immobilized on the sensor surface, as previously reported^23^. First, the sensors were treated with O_2_ plasma for 1 min to form OH groups on the glass substrate. After the O_2_ plasma treatment, the sensors were treated with 10% (3-aminopropyl) triethoxysilane (Sigma, St. Louis, MO, USA) for 2 h at room temperature, followed by washing with 10 mM sulfuric acid for 15 min and then with distilled water. Subsequently, the sensors were treated with 10 mM 3,3′-dithiopropionic acid for 2 h at room temperature, followed by washing with distilled water. To activate the carboxylic groups, the sensors were treated with 12.9 mM *N*-3-dimethylaminopropyl (Sigma) and 8.7 mM *N*-hydroxysulfosuccinimide (Sigma) for 1 h, and then washed with ethanol. Finally, the sensors were incubated in a solution of 3 μM amine-modified aptamer for 6 h and washed thoroughly with distilled water.

### Antibiotic coating on the sensor surface

For each antimicrobial agent, a stock solution of 1 mg/ml was prepared in ultra-pure water, and diluted in the growth medium. Ten microliters of the antimicrobial solutions were added to the wells of the Gram-negative and Gram-positive chips. They were then dried using a freeze-dryer.

### Real-time capacitance measurements

Subcultured bacterial colonies were released to growth media and the suspension was diluted to a concentration of 10^5^ CFU/ml for the AST experiments. 200 μl of bacterial solution was added to each well of the *e*-AST chip. Then, the *e*-AST chip was mounted on a capacitance impedance analyzer (Proteomtech Inc., Seoul, South Korea) capable of simultaneously measuring the capacitance of 60 sensors (see Supplementary Fig. S1). It was then placed inside an incubator maintained at 37 °C. The capacitance was measured with a peak-to-peak alternating current signal of 10 mV at a frequency of 1,000 Hz. The capacitance impedance analyzer inside the incubator was linked via Bluetooth to the computer, and the data from each sensor were collected every 16.5 min.

### Broth microdilution test

For the BMD tests, antimicrobial solutions were prepared from the stock solution. Antimicrobial agents at the appropriate concentration were pipetted into the wells of BD Falcon™ 96 MicroWell plates (BD Biosciences, San Jose, CA, USA) at a volume of 200 μl, and the wells were then inoculated with 10 μl bacterial stock solution, each at a final concentration of 5 × 10^5^ CFU/ml^31^. After 16 h of incubation at 37 °C, the MIC values of the microdilution wells were read as the concentration, at which more than 80% reduction in growth was observed, as compared to that in the control by unaided visual inspection^5,32^.

### Estimation of similarity measure

A similarity measure was estimated using a pattern-matching algorithm^33^. Briefly, the complexity of the (Δ*C/C*_0_) × *d*(Δ*C/C*_0_)/*dt* curves was reduced using principal component analysis, and the feature of each curve was extracted via linear discriminant analysis. Then, the similarity measure was determined by *a*/(*a* + *b*), where *a* and *b* are the Euclidean distance to the features of cell-free media curve and the positive control curves, respectively. For cell-free media and the positive control, the similarity measure was expected to be 0 and 1, respectively. The program was coded using MATLAB.

### Statistical analysis

The 95% confidence intervals for the proportion of CA, including mE, ME, and VME between the *e*-AST, VITEK 2 Systems, and gold standard BMD method, were also calculated.

## Results and discussion

### Relationship between real-time capacitance and bacterial growth

**Figure 2:**
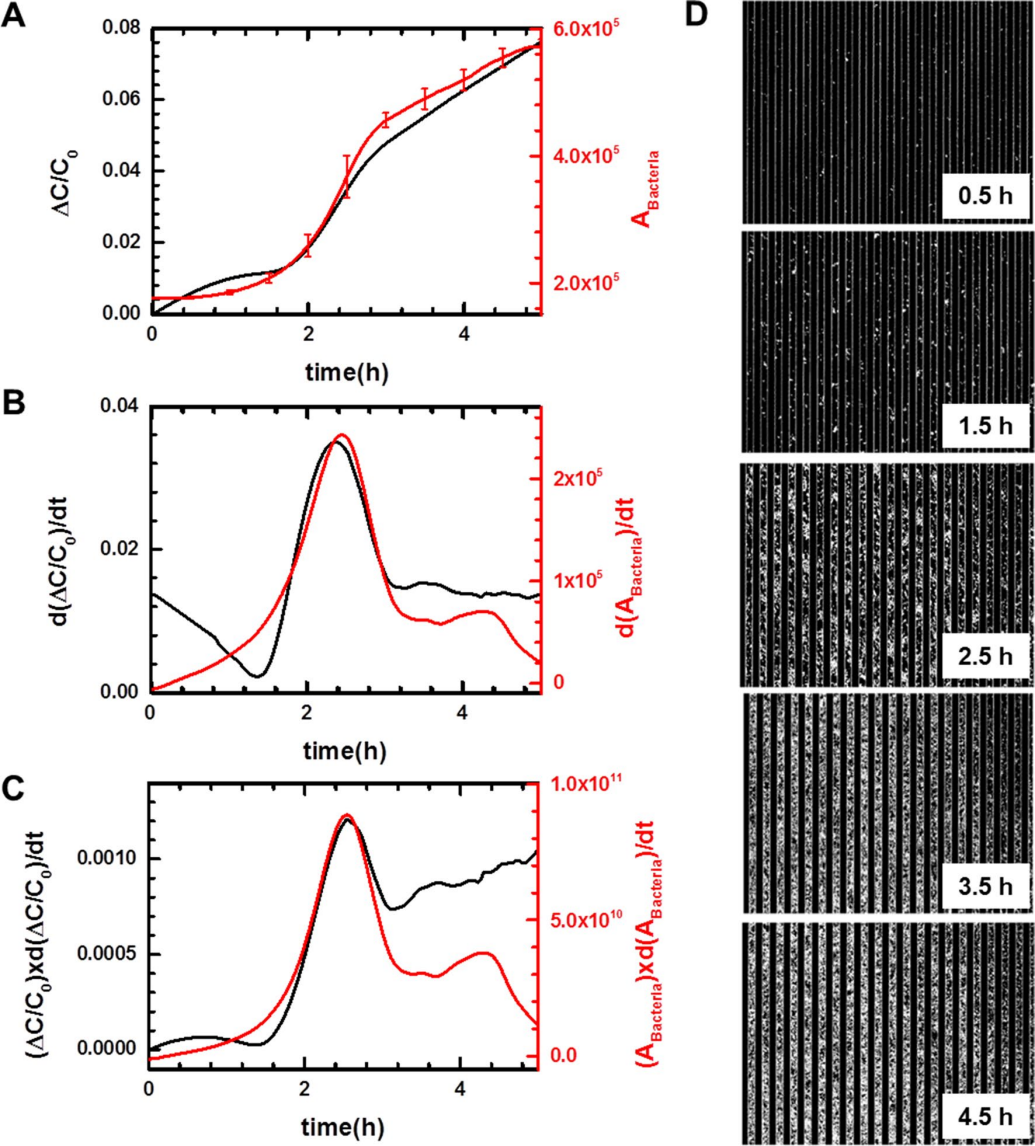
(**A**) Time dependence of normalized capacitance change (Δ*C*/*C*_*0*_, black curve) and the area occupied by bacteria (*A*_Bacteria_, red curve) when *E. coli* U433 was cultured. (**B**) *d*(Δ*C*/*C*_*0*_)/*dt* (black curve) and *d*(*A*_Bacteria_)/*dt* (red curve) numerically calculated from the data shown in (**A**). (**C**) (Δ*C*/*C*_*0*_) × *d*(Δ*C*/*C*_*0*_)/*dt* (black curve) and (*A*_Bacteria_) × *d*(*A*_Bacteria_)/*dt* (red curve). (**D**) Time-lapse phase contrast optical images acquired when *E. coli* U433 was cultured in a Chamlide chamber.

Prior to the AST test, we measured the real-time capacitance when *E. coli* U433 of 10^5^ CFU/ml was cultured in media (black curve, Fig. 2A), where *C*_0_ is the initial capacitance value and *ΔC* = *C − C*_0_. The capacitance increased slowly from 0 to 2 h, and rapidly from 2 to 2.9 h; thereafter, it increased steadily. Accordingly, the capacitance increase rate, calculated numerically by differentiating the capacitance with respect to time, *d*(Δ*C*/*C*_0_)/*dt*, shows a peak at 2.4 h (black curve, Fig. 2B). To investigate the relationship between the real-time capacitance and the number of bacteria bound to the sensor surface via aptamers, phase contrast optical images were acquired when *E. coli* U433 at 10^5^ CFU/ml was cultured in a Chamlide chamber (Live Cell Instrument, Inc., Seoul, South Korea) maintained at 37 °C (Fig. 2D).

The area occupied by bacteria (*A*_Bacteria_) was estimated using MATLAB built-in function, which is used to detect the edge line of cells, as a function of time (red curve, Fig. 2A). As for the capacitance, *A*_Bacteria_ exhibited three regions depending on the increasing rate. When *A*_Bacteria_ was differentiated with respect to time, *d*(*A*_Bacteria_)/*dt* exhibited a peak at the same time as *d*(Δ*C*/*C*_0_)/*dt* (Fig. 2B), indicating that bacterial growth is closely related to real-time capacitance. In particular, when *d*(Δ*C*/*C*_0_)/*dt* and *d*(*A*_Bacteria_)/*dt* were multiplied by Δ*C*/*C*_0_ and *A*_Bacteria_, respectively, similar behaviors were observed before 3.5 h (Fig. 2C). Therefore, the capacitance data were analyzed in terms of (Δ*C*/*C*_0_) × *d*(Δ*C*/*C*_0_)/*dt*.

### Determination of antimicrobial susceptibility using real-time capacitance measurements

**Figure 3:**
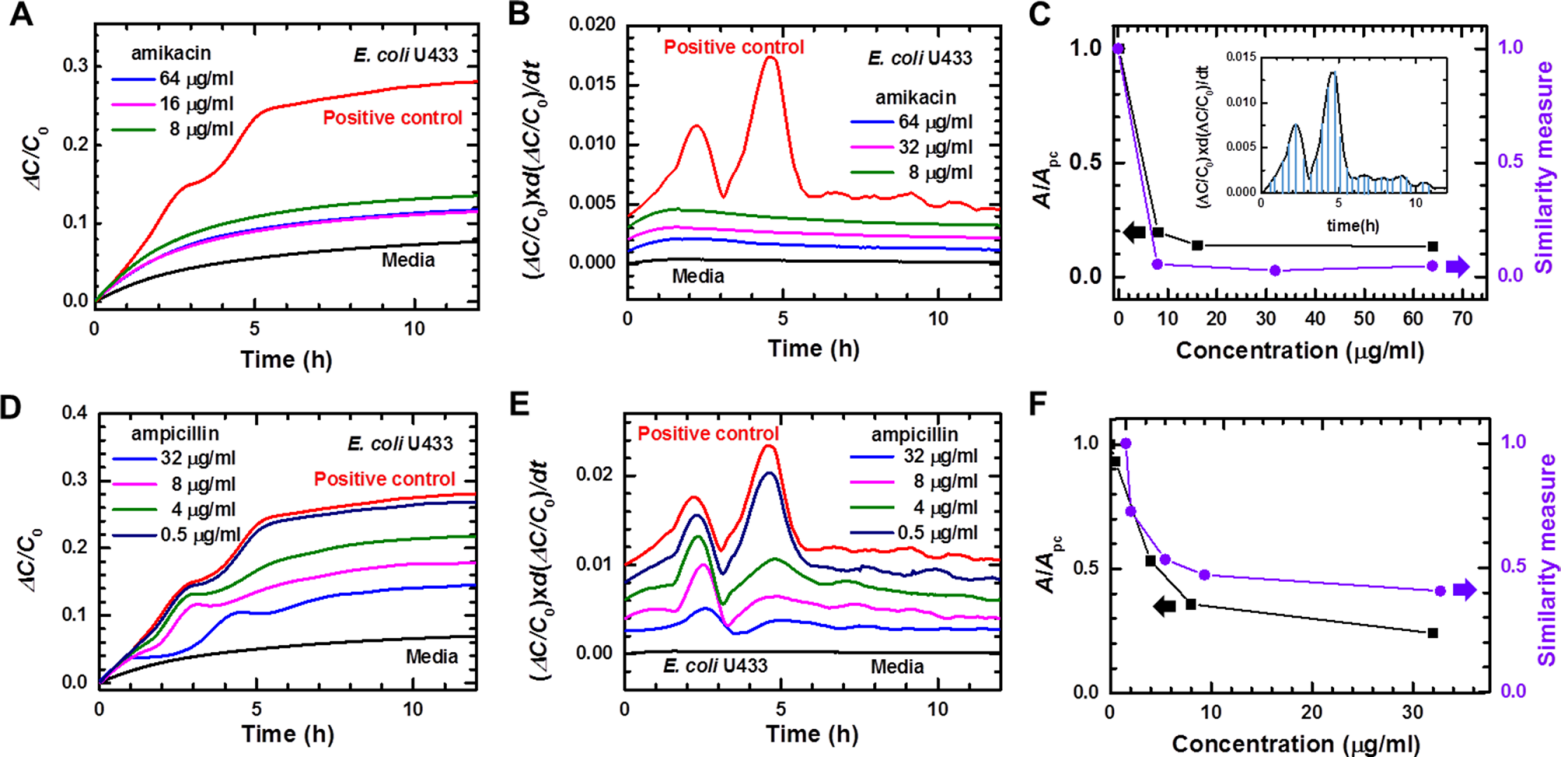
Time dependence of Δ*C/C*_0_ measured for *E. coli* U433 treated with different concentrations of (**A**) amikacin and (**D**) ampicillin. Positive control represents the data obtained from *E. coli* U 433 without antibiotics, and media represents the data of cell-free media. (Δ*C/C*_0_) × *d*(Δ*C/C*_0_)/*dt* numerically calculated for *E. coli* U433, which was treated with different concentrations of (**B**) amikacin and (**E**) ampicillin. The (Δ*C/C*_0_) × *d*(Δ*C/C*_0_)/*dt* curves are shifted to facilitate viewing. The area enclosed by the (Δ*C/C*_0_) × *d*(Δ*C/C*_0_)/*dt* curve and the X-axis, which is denoted by the shaded region in the inset of (**C**), normalized by the area of positive control (*A*_pc_) (*A*/*A*_pc_, black symbols) and similarity measures (purple symbols) estimated using the pattern matching algorithm for *E. coli* U433, which was treated with different concentrations of (**C**) amikacin and (**F**) ampicillin.

To investigate whether real-time capacitance measurements can be employed to perform ASTs, we measured the real-time capacitance for *E. coli* U433 treated with different concentrations of amikacin (Fig. 3A) and ampicillin (Fig. 3D), and then calculated (Δ*C*/*C*_0_) × *d*(Δ*C*/*C*_0_)/*dt* (Fig. 3B, E). For amikacin, (Δ*C*/*C*_0_) × *d*(Δ*C*/*C*_0_)/*dt* showed no peaks at all concentrations, indicating that *E. coli* U433 is susceptible to amikacin. In contrast, peaks were observed at all concentrations of ampicillin, indicating that U433 is resistant to ampicillin. To determine the antimicrobial susceptibility more quantitatively, we calculated the area enclosed by the curve of (Δ*C*/*C*_0_) × *d*(Δ*C*/*C*_0_)/*dt* and the *x*-axis (inset of Fig. 3C). For all concentrations of amikacin, *A*/*A*_*pc*_ was smaller than 0.2, where *A*_*pc*_ is the area estimated for the positive control (black symbols, Fig. 3C). In contrast, *A*/*A*_*pc*_ was higher than 0.2 for all concentrations of ampicillin (black symbols, Fig. 3F). In addition to *A*/*A*_*pc*_, we estimated a similarity measure using a pattern-matching algorithm, which shows the closeness of (Δ*C*/*C*_0_) × *d*(Δ*C*/*C*_0_)/*dt* curve obtained in the presence of antimicrobial agents to that of positive control (purple symbols, Fig. 3C, F). The similarity measure was lower than 0.1 for amikacin and higher than 0.4 for ampicillin. The comparison with the results obtained from the gold standard BMD tests and VITEK 2 systems (see Supplementary Table S2) suggests that the cutoff values between bacterial growth and inhibition are approximately 0.2 and 0.4 for *A*/*A*_*pc*_ and the similarity measure, respectively. *A*/*A*_*pc*_ and the similarity measure exhibited similar behaviors; however, in comparison with the *A*/*A*_*pc*_, the similarity measure provides a clearer distinction between bacterial growth and inhibition. Therefore, the similarity measure is adopted for the proposed *e*-AST chips.

**Figure 4:**
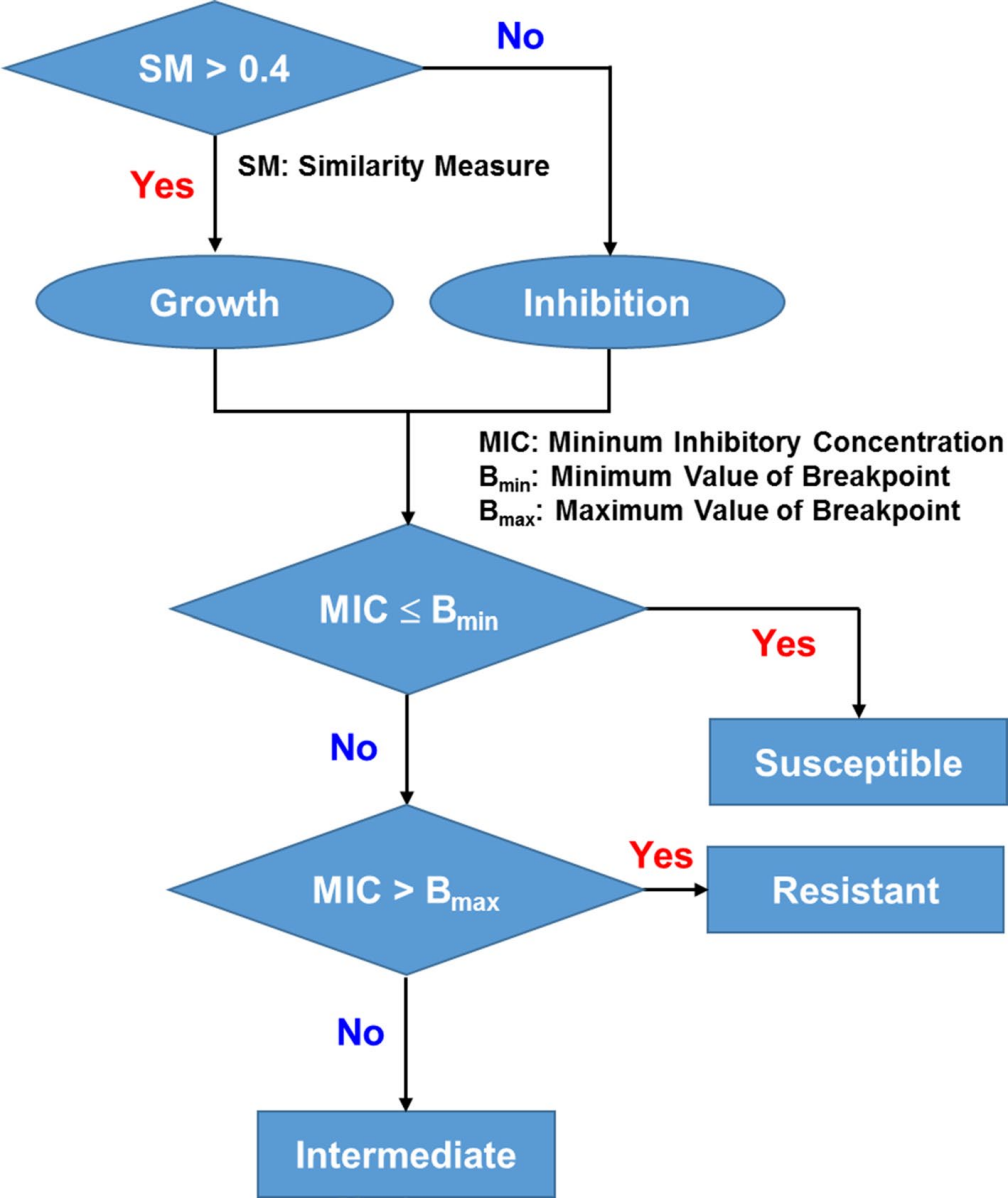
Data interpretation and susceptibility determination. SM denotes similarity measure.

The antimicrobial susceptibility was determined, as shown in Fig. 4. Assuming that the cutoff of similarity measure between bacterial growth and inhibition is 0.4, the MIC was estimated to be less than 8 μg/ml for amikacin. It is lower than the breakpoint of 8 – 16 μg/ml suggested by CLSI; therefore, *E. coli* U433 was determined to be susceptible to amikacin. For ampicillin, the MIC was estimated to be greater than 32 μg/ml, higher than the breakpoint of 2 – 8 μg/ml, indicating that *E. coli* U433 is resistant to ampicillin. The gold standard BMD tests and VITEK 2 systems also showed that *E. coli* U433 is susceptible to amikacin and resistant to ampicillin, indicating that antimicrobial susceptibility can be determined using the *e*-AST system.

### Performance of *e*-AST system in clinical samples

**Figure 5:**
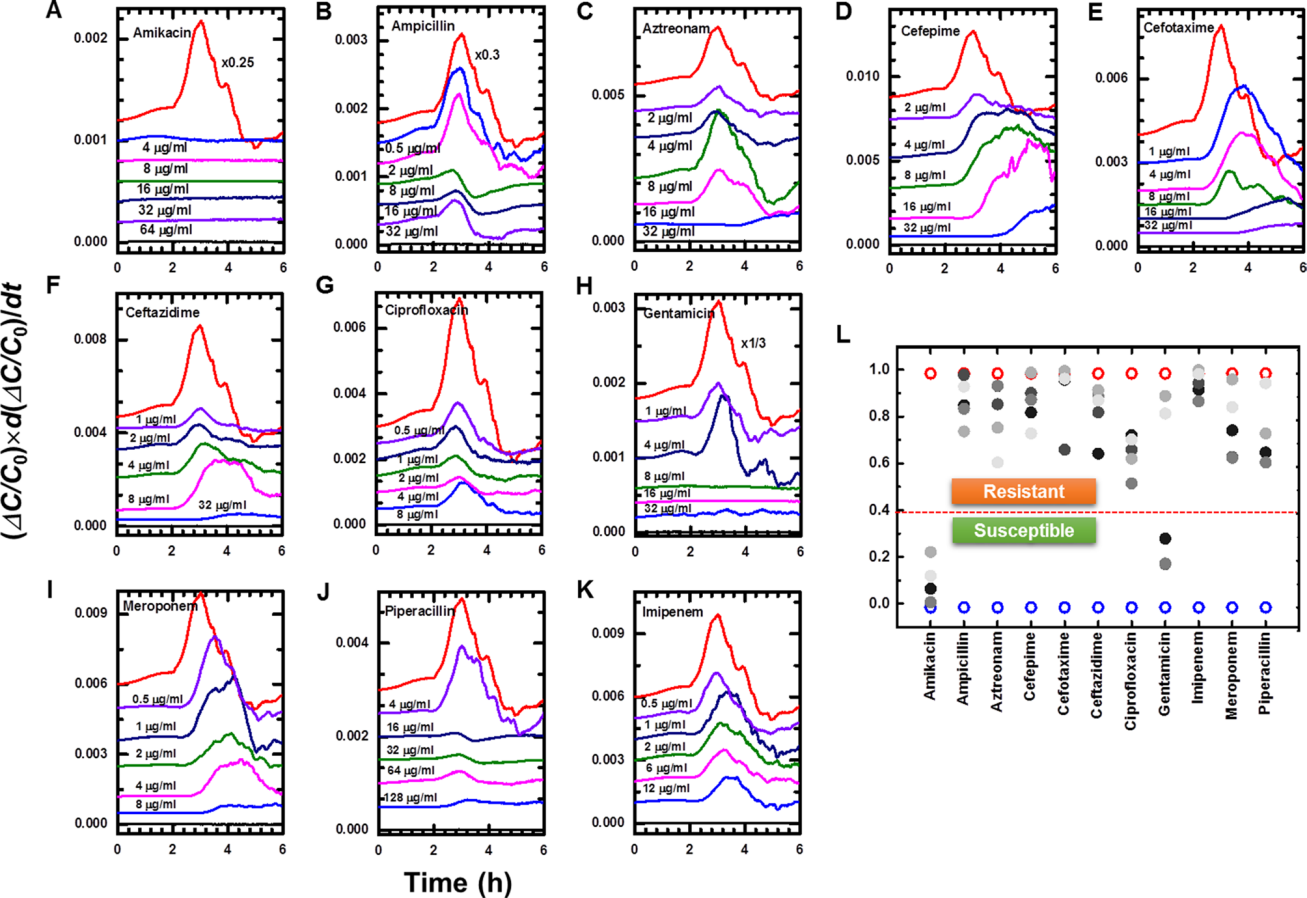
(**A**–**K**) Time dependence of (Δ*C/C*_0_) × *d*(Δ*C/C*_0_)/*dt* obtained using Gram-negative *e*-AST chip for *A. baumannii* R4197. The (Δ*C/C*_0_) × *d*(Δ*C/C*_0_)/*dt* curves are shifted to facilitate viewing. Positive control (red line) represents the data obtained from *A. baumannii* R4197 without antibiotics, and media (black line) represents the data of cell-free media. (**L**) Similarity measures estimated using the pattern matching algorithm for *A. baumannii* R4197. Higher concentration is indicated by the darker color. Blue and red circles indicate the similarity measure of cell-free media and *A. baumannii* R4197 without antibiotics, respectively.

For the AST tests, Gram-negative and Gram-positive chips were prepared. The list of antibiotics and their concentrations is presented in Table S1, which was selected from VITEK 2 kits used at Yonsei University Severance Hospital. To evaluate the performance of the *e*-AST system, five bacterial isolates per strain were tested for four Gram-negative strains (*E. coli*, *A. baumannii*, *P. aeruginosa*, and *K. pneumoniae*) and two Gram-positive strains (*S. aureus* and *E. faecalis*) (Table 1). Figure 5A – K show the typical results measured for *A. baumannii* R4197 using the Gram-negative *e*-AST chip. The data were monitored for 6 h because the time dependence data (Fig. 3) revealed that antimicrobial susceptibility could be determined within 6 h.

From the capacitance measured as a function of time, (Δ*C*/*C*_0_) × *d*(Δ*C*/C_0_)/*dt* was numerically calculated, and the similarity measures were estimated using a pattern-matching algorithm (Fig. 5L). The antimicrobial susceptibilities determined by the flow chart in Fig. 4 are summarized in Table 2 for various antibiotics. *A. baumannii* R4197 was determined to be susceptible to amikacin and gentamicin, and resistant to other antibiotics. These results are consistent with the BMD results, supporting the application possibility of *e*-AST chips to perform ASTs.

**Table 2:**
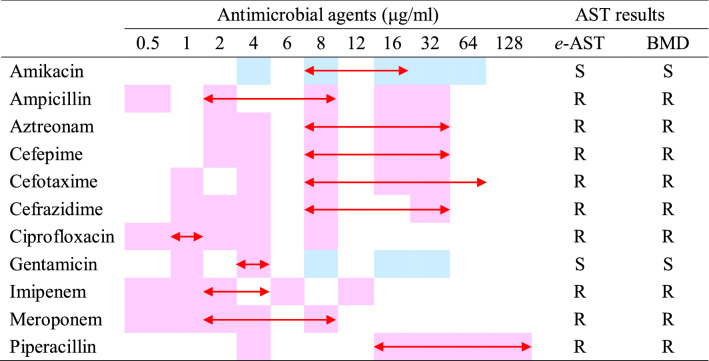
The *e*-AST results for *A. baumannii* R4197 and the comparison with gold standard BMD.

For the *e*-AST and BMD test: SM (similarity measure) < 0.4, blue color; SM > 0.4, pink color; red arrow, MIC interpretive criterion (μg/mL); S, susceptible; R, resistant.

To estimate the error rates of the *e*-AST system, a total of 4,554 tests were performed and discrepancies were determined by comparing the results of the *e*-AST chips with those of the BMD test as the reference (Table 3 and see Supplementary Table S3). According to the guidance from the FDA, the discrepancies were classified as minor error, major error, very major error, and category agreement (CA). Minor errors (mEs) represent the strains that were interpreted as “intermediate” using the *e*-AST chip but were determined to be either sensitive or resistant by the reference test. Major errors (MEs) represent the strains that were interpreted as “resistant” using the *e*-AST chip but were determined to be sensitive by the reference test. Very major errors (VMEs) represent the strains that were interpreted as “sensitive” using the *e*-AST chip but were determined to be resistant by the reference test. CA is the same category of rates (S, I, R) provided by the *e*-AST chip and the reference test. For all strains, CA was higher than 95%, and mEs, MEs, and VMEs rates were lower than 5%, 1%, and 1.8%, respectively. For *P. aeruginosa*, 187 tests were conducted and there were 4 mEs, 1 ME, and 1 VME. The ME and VME occurred in ceftazidime and imipenem, respectively (see Supplementary Table S3).

**Table 3:** Discrepancy rate using clinical isolates.

Bacteria	No. Test	Discrepancy number			Discrepancy rate (%)			
		mEs	MEs	VMEs	mEs	MEs	VMEs	CA
**Gram negative**								
*E. coli*	187	3	2	0	1.60	1.07	0	97.33
*A. baumannii*	165	4	0	0	2.42	0	0	97.58
*P. aeruginoa*	187	4	1	1	2.14	0.53	0.53	96.79
*K. pneumoniae*	176	3	1	0	1.70	0.57	0	97.73
	715	14	4	1	1.96	0.56	0.14	97.34
**Gram positive**								
*S. aureus*	165	0	0	3	0	0	1.82	98.18
*E. faecalis*	165	9	0	0	5.46	0	0	94.55
	330	9	0	3	2.73	0	0.91	96.37
Total	1,045	23	4	4	2.20	0.38	0.38	97.03

The *e*-AST results were compared with the BMD results to calculate the discrepancy rates. For *e*-AST: *mE* minor error, *ME* major error, *VME* very major error, *CA* categorical agreement.

In the case of *S. aureus*, there were 3 VMEs among 165 tests. The 3 VMEs were from *S. aureus* (R4319) treated with clindamycin (see Supplementary Table S3). In the case focusing on the six β-lactam resistant strains composed of ESBL, corresponding to 220 combinations of antibiotic-bacteria tests, we observed 216 (98.18%) CA, 2 (0.96%) mEs, 2 (0.96%) MEs, and 0 (0%) VME (Table 4). Overall, the total CA, mEs, MEs, and VMEs rates were 97.03%, 2.20%, 0.38%, and 0.38%, respectively. These values satisfy the CLSI recommendations, in which the acceptable inter-method VMEs, MEs, and mEs rates are 1.5%, 3%, and 10%, respectively^34^.

**Table 4:** Discrepancy rate of two representative ESBL-positive isolates from sepsis patients.

ESBL-bacteria	No. strains	No. test	Discrepancy number			Discrepancy rate (%)			
			mEs	MEs	VMEs	mEs	MEs	VMEs	CA
*E. coli*	2	77	1	0	0	1.29	0	0	98.70
*K. pneumoniae*	4	143	1	2	0	2.09	0.69	0	97.20

Extended-spectrum β-lactamase (ESBL) positive strains.

## Conclusion

We developed *e*-AST systems based on an array of 60 aptamer-functionalized capacitance sensors and a pattern-matching algorithm for rapid AST. Bacterial behaviors, such as growth and inhibition, were monitored in real-time by measuring the capacitance change, thereby allowing the determination of antimicrobial susceptibility within 6 h, which is shorter in comparison with that by the conventional methods (~ 16 h). To validate the clinical usefulness of *e*-AST, the *e*-AST results were compared with the results of gold standard BMD for six clinical strains from septic patients, including ESBL-positive *E. coli* and *K. pneumoniae* as antibiotic-resistant pathogens. The discrepancies between *e*-AST and BMD tests were estimated to be 2.20% mE, 0.38% ME, and 0.38%, which are lower than the FDA requirements (mE ≤ 10%, ME ≤ 3%, and VME ≤ 1.5%). These results demonstrate that *e*-AST can be applied for rapid AST. Diagnosis of sepsis by *e*-AST may cost more than gold standard method; however, it is expected that *e*-AST system could increase survival rate of sepsis patients.

## Supplementary information

Supplementary file1
