# Supplementary material for: Electrical antimicrobial susceptibility testing based on aptamer-functionalized capacitance sensor array for clinical isolates
Source: Sci Rep. 2020 Aug 13;10:13709. doi: 10.1038/s41598-020-70459-3 (PMC7426404; doi:10.1038/s41598-020-70459-3)
Supplement: Supplementary file 1 — Supplementary file1 [file 41598_2020_70459_MOESM1_ESM.docx]

**Electrical Antimicrobial Susceptibility Testing Based on**

**Aptamer-Functionalized Capacitance Sensor Array for Clinical Isolates**

**– Supplementary Material**

Kyo-Seok Lee^1^, Sun-Mi Lee^2*^, Jeseung Oh^3^, In Ho Park^4,5^, Jun Ho Song^1^, Myeonggil Han^4^, Dongeun Yong^6^, Kook Jin Lim^2,3^, Jeon-Soo Shin^2,4,5*^, Kyung-Hwa Yoo^1,2*^

^1^Department of Physics, Yonsei University, Seoul 03722, Republic of Korea

^2^Nanomedical Graduate Program, Yonsei University, Seoul 03722, Republic of Korea

^3^Proteomtech Inc., 1101 Wooree-Venture Town, Seoul 07573, Republic of Korea

^4^Department of Microbiology, Yonsei University, College of Medicine, Seoul 03722, Republic of Korea

^5^Severance Biomedical Science Institute and Institute for Immunology and Immunological Diseases, Yonsei University College of Medicine, Seoul 03722, Republic of Korea

^6^Department of Laboratory Medicine and Research Institute of Bacterial Resistance, Yonsei University College of Medicine, Seoul 03722, Republic of Korea

Correspondence and requests for materials should be addressed to S.M.L. (E-mail: [sunmilee@yonsei.ac.kr](mailto:sunmilee@yonsei.ac.kr)), J.S.S (E-mail: [jsshin6203@yuhs.ac](mailto:jsshin6203@yuhs.ac)), or K.H.Y. (E-mail: [khyoo@yonsei.ac.kr](mailto:khyoo@yonsei.ac.kr))


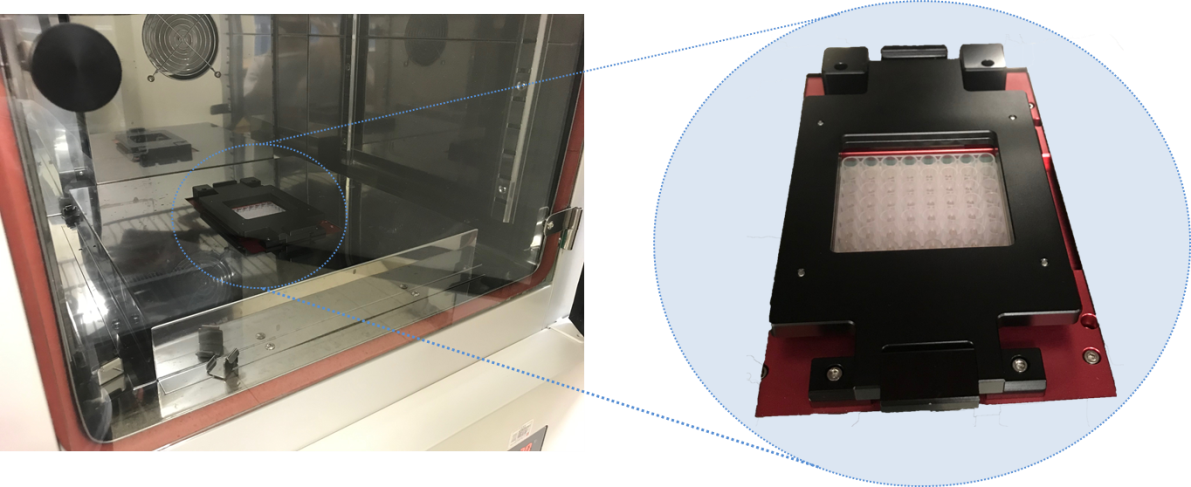


**Figure S1. Photograph of the *e*-AST measurement system.** A 60 channels array mounted in an incubator maintained at 37 °C.

**Table S1.** The list of antimicrobial agents in *e*-AST chip

| Antimicrobial agents | | Concentration (μg/ml) | | | | |
| --- | --- | --- | --- | --- | --- | --- |
| Gram-negative  *e*-AST chip | Amikacin | 64 | 32 | 16 | 8 | 4 |
|  | Ampicillin | 32 | 16 | 8 | 2 | 0.5 |
|  | Aztreonam | 32 | 16 | 8 | 4 | 2 |
|  | Cefepime | 32 | 16 | 8 | 4 | 2 |
|  | Cefotaxime | 32 | 16 | 8 | 4 | 1 |
|  | Ceftazidime | 32 | 16 | 8 | 4 | 1 |
|  | Ciprofloxacin | 8 | 4 | 2 | 1 | 0.5 |
|  | Gentamicin | 32 | 16 | 4 | 2 | 1 |
|  | Imipenem | 12 | 6 | 2 | 1 | 0.5 |
|  | Meroponem | 8 | 4 | 2 | 1 | 0.5 |
|  | Piperacillin | 128 | 64 | 32 | 16 | 4 |
| Gram-positive  *e*-AST chip | Ampicillin | 32 | 16 | 8 | 2 | 0.5 |
|  | Ciprofloxacin | 8 | 4 | 2 | 1 | 0.5 |
|  | Clindamycin | 8 | 4 | 2 | 1 | 0.25 |
|  | Erythromycin | 8 | 4 | 2 | 1 | 0.5 |
|  | Gentamicin | 32 | 16 | 8 | 4 | 1 |
|  | Linezolid | 8 | 4 | 2 | 1 | 0.5 |
|  | Mupirocin | 8 | 4 | 2 | 1 | 0.5 |
|  | Oxacillin | 8 | 4 | 2 | 1 | 0.5 |
|  | Teicoplanin | 16 | 8 | 4 | 1 | 0.5 |
|  | Tetracycline | 8 | 2 | 1 | 0.5 | 0.25 |
|  | Vancomycin | 16 | 8 | 4 | 2 | 1 |

**Table S2**. The comparison of *e*-AST results in *E. coli* U 433 with gold standard BMD and clinical VITEK 2

| Antimicrobial | μg/ml | *e*-AST | | BMD | VITEK^®^ 2 | |
| --- | --- | --- | --- | --- | --- | --- |
|  |  | *A*/*A*_pc_ | SM | Result | Result | MIC (μg/ml) |
| Amikacin | 64 | 0.13 | 0.05 | S | S | 4 |
|  | 16 | 0.14 | 0.03 |  |  |  |
|  | 8 | 0.20 | 0.06 |  |  |  |
| Ampicillin | 32 | 0.24 | 0.41 | R | R | ≥ 32 |
|  | 8 | 0.36 | 0.47 |  |  |  |
|  | 4 | 0.53 | 0.53 |  |  |  |
|  | 0.5 | 0.93 | 0.73 |  |  |  |

Each test was performed in triplicate. SM, similarity measure; S, susceptible; R, resistant.

**Table S3**. The comparison of *e*-AST results with gold standard BMD and clinical VITEK 2

| ***E.coli* (U433)** | | | | | | | |
| --- | --- | --- | --- | --- | --- | --- | --- |
| Antibiotic (μg/ml) | *e*-AST | | | | BMD | Vitek | MIC (μg/ml) |
|  | Test 1 | Test 2 | Test 3 | Test 4 |  |  |  |
| Amikacin | S | S | S | S | S | S | ≤ 2 |
| Ampicillin | R | R | R | R | R | R | ≥ 32 |
| Aztreonam | S | S | S | S | S | S | ≤ 1 |
| Cefepime | S | S | S | S | S | S | ≤ 1 |
| Cefotaxime | S | S | S | S | S | S | ≤ 4 |
| Ceftazidime | S | S | S | S | S | S | ≤ 1 |
| Ciprofloxacin | R | S | S | S | S | - | - |
| Gentamicin | I | R | S | S | S | S | ≤ 1 |
| Imipenem | S | S | S | S | S | - | - |
| Meroponem | S | S | S | S | S | - | - |
| Piperacillin | R | R | R | R | R |  | - |

For the *e*-AST, BMD and VITEK 2 test: S, susceptible; I, intermediate; R, resistant.

| ***E.coli* (U556) - ESBL** | | | | | | | |
| --- | --- | --- | --- | --- | --- | --- | --- |
| Antibiotic (μg/ml) | *e*-AST | | | | BMD | Vitek | MIC (μg/ml) |
|  | Test 1 | Test 2 | Test 3 | Test 4 |  |  |  |
| Amikacin | S | S | S | S | S | S | 4 |
| Ampicillin | R | R | R | R | R | R | ≥ 32 |
| Aztreonam | R | R | R | R | R | R | ≥ 64 |
| Cefepime | R | R | R | R | R | R | ≥ 64 |
| Cefotaxime | R | R | R | R | R | R | ≥ 64 |
| Ceftazidime | R | R | R | R | R | R | 16 |
| Ciprofloxacin | R | R | R | R | R | - | - |
| Gentamicin | R | R | R | I | R | R | ≥ 16 |
| Imipenem | S | S | S | S | S | - | - |
| Meropenem | S | S | S | S | S | - | - |
| Piperacillin | R | R | R | R | R | - | - |

For the *e*-AST, BMD and VITEK 2 test: S, susceptible; I, intermediate; R, resistant.

| ***E.coli* (B12327)** | | | | | | |
| --- | --- | --- | --- | --- | --- | --- |
| Antibiotic (μg/ml) | *e*-AST | | | BMD | Vitek | MIC (μg/ml) |
|  | Test 1 | Test 2 | Test 3 |  |  |  |
| Amikacin | S | S | S | S | S | ≤ 2 |
| Ampicillin | S | S | S | S | S | 8 |
| Aztreonam | S | S | S | S | S | ≤ 1 |
| Cefepime | S | S | S | S | S | ≤ 1 |
| Cefotaxime | S | S | S | S | S | ≤ 1 |
| Ceftazidime | S | S | S | S | S | ≤ 0.25 |
| Ciprofloxacin | S | S | S | S | - | - |
| Gentamicin | S | S | S | S | S | ≤ 1 |
| Imipenem | S | S | S | S | - | - |
| Meropenem | S | S | S | S | - | - |
| Piperacillin | S | S | S | S | - | - |

For the *e*-AST, BMD and VITEK 2 test: S, susceptible; I, intermediate; R, resistant.

| ***E.coli* (U5307)** | | | | | | |
| --- | --- | --- | --- | --- | --- | --- |
| Antibiotic (μg/ml) | *e*-AST | | | BMD | Vitek | MIC (μg/ml) |
|  | Test 1 | Test 2 | Test 3 |  |  |  |
| Amikacin | S | S | S | S | S | ≤ 2 |
| Ampicillin | R | I | R | R | R | ≥ 32 |
| Aztreonam | S | S | S | S | S | ≤ 1 |
| Cefepime | S | S | S | S | S | ≤ 1 |
| Cefotaxime | S | S | S | S | S | ≤ 1 |
| Ceftazidime | S | S | S | S | S | ≤ 0.25 |
| Ciprofloxacin | S | S | S | S | - | - |
| Gentamicin | S | S | S | S | S | ≤ 1 |
| Imipenem | S | S | S | S | - | - |
| Meropenem | S | S | S | S | - | - |
| Piperacillin | S | S | S | S | - | - |

For the *e*-AST, BMD and VITEK 2 test: S, susceptible; I, intermediate; R, resistant.

| ***E.coli* (U6267)-ESBL** | | | | | | |
| --- | --- | --- | --- | --- | --- | --- |
| Antibiotic (μg/ml) | *e*-AST | | | BMD | Vitek | MIC (μg/ml) |
|  | Test 1 | Test 2 | Test 3 |  |  |  |
| Amikacin | S | S | S | S | S | ≤ 2 |
| Ampicillin | R | R | R | R | R | ≥ 32 |
| Aztreonam | R | R | R | R | S | ≤ 1 |
| Cefepime | R | R | R | R | S | ≤ 1 |
| Cefotaxime | S | S | S | S | S | ≤ 1 |
| Ceftazidime | S | S | S | S | R | ≥ 64 |
| Ciprofloxacin | S | S | S | S | - | - |
| Gentamicin | S | S | S | S | R | ≥ 16 |
| Imipenem | S | S | S | S | - | - |
| Meropenem | S | S | S | S | - | - |
| Piperacillin | R | R | R | R | - | - |

For the *e*-AST, BMD and VITEK 2 test: S, susceptible; I, intermediate; R, resistant.

| ***A. baumannii* (R4197)** | | | | | | |
| --- | --- | --- | --- | --- | --- | --- |
| Antibiotic (μg/ml) | *e*-AST | | | BMD | Vitek | MIC (μg/ml) |
|  | Test 1 | Test 2 | Test 3 |  |  |  |
| Amikacin | S | S | S | S | S | - |
| Ampicillin | R | R | R | R | - | - |
| Aztreonam | R | R | R | R | - | - |
| Cefepime | R | R | R | R | R | ≥ 64 |
| Cefotaxime | R | R | R | R | R | ≥ 64 |
| Ceftazidime | R | R | R | R | R | ≥ 64 |
| Ciprofloxacin | R | R | R | R | R | ≥ 4 |
| Gentamicin | S | S | S | S | S | 4 |
| Imipenem | R | R | R | R | R | ≥ 16 |
| Meropenem | R | R | R | R | R | ≥ 16 |
| Piperacillin | R | R | R | R | R | ≥ 128 |

For the *e*-AST, BMD and VITEK 2 test: S, susceptible; I, intermediate; R, resistant.

| ***A. baumannii* (R4356)** | | | | | | |
| --- | --- | --- | --- | --- | --- | --- |
| Antibiotic (μg/ml) | *e*-AST | | | BMD | Vitek | MIC (μg/ml) |
|  | Test 1 | Test 2 | Test 3 |  |  |  |
| Amikacin | R | R | R | R | R | - |
| Ampicillin | R | R | R | R | - | - |
| Aztreonam | R | R | R | R | - | - |
| Cefepime | R | R | R | R | R | ≥ 64 |
| Cefotaxime | R | R | R | R | R | ≥ 64 |
| Ceftazidime | R | R | R | R | R | ≥ 64 |
| Ciprofloxacin | R | R | R | R | R | ≥ 4 |
| Gentamicin | R | R | R | R | S | ≥ 16 |
| Imipenem | R | R | R | R | R | ≥ 16 |
| Meropenem | R | R | R | R | R | ≥ 16 |
| Piperacillin | R | I | I | R | R | ≥ 128 |

For the *e*-AST, BMD and VITEK 2 test: S, susceptible; I, intermediate; R, resistant.

| ***A. baumannii* (P762)** | | | | | | |
| --- | --- | --- | --- | --- | --- | --- |
| Antibiotic (μg/ml) | *e*-AST | | | BMD | Vitek | MIC (μg/ml) |
|  | Test 1 | Test 2 | Test 3 |  |  |  |
| Amikacin | S | S | S | S | S | - |
| Ampicillin | R | R | R | R | - | - |
| Aztreonam | R | R | R | R | - | - |
| Cefepime | R | R | R | R | S | 2 |
| Cefotaxime | R | R | R | R | S | 8 |
| Ceftazidime | R | R | R | R | S | 4 |
| Ciprofloxacin | S | S | S | S | S | ≤ 0.25 |
| Gentamicin | S | S | S | S | S | ≤ 1 |
| Imipenem | S | S | S | S | S | ≤ 1 |
| Meropenem | S | S | S | S | S | ≤ 1 |
| Piperacillin | R | R | R | R | S | 8 |

For the *e*-AST, BMD and VITEK 2 test: S, susceptible; I, intermediate; R, resistant.

| ***A. baumannii* (T800)** | | | | | | |
| --- | --- | --- | --- | --- | --- | --- |
| Antibiotic (μg/ml) | *e*-AST | | | BMD | Vitek | MIC (μg/ml) |
|  | Test 1 | Test 2 | Test 3 |  |  |  |
| Amikacin | R | R | R | R | R | - |
| Ampicillin | R | R | R | R | - | - |
| Aztreonam | R | R | R | R | - | - |
| Cefepime | R | R | R | R | R | 2 |
| Cefotaxime | R | R | R | R | R | 8 |
| Ceftazidime | R | R | R | R | R | 4 |
| Ciprofloxacin | R | R | R | R | R | ≤ 0.25 |
| Gentamicin | R | R | R | R | R | ≤ 1 |
| Imipenem | R | R | R | R | R | ≥ 16 |
| Meropenem | R | R | R | R | R | ≥ 16 |
| Piperacillin | R | R | R | R | R | ≥ 128 |

For the *e*-AST, BMD and VITEK 2 test: S, susceptible; I, intermediate; R, resistant.

| ***A. baumannii* (R4299)** | | | | | | |
| --- | --- | --- | --- | --- | --- | --- |
| Antibiotic (μg/ml) | *e*-AST | | | BMD | Vitek | MIC (μg/ml) |
|  | Test 1 | Test 2 | Test 3 |  |  |  |
| Amikacin | R | R | R | R | R | - |
| Ampicillin | R | R | R | R | - | - |
| Aztreonam | I | R | R | R | - | - |
| Cefepime | I | R | R | R | R | ≥ 64 |
| Cefotaxime | R | R | R | R | R | ≥ 64 |
| Ceftazidime | R | R | R | R | R | ≥ 64 |
| Ciprofloxacin | R | R | R | R | R | ≥ 4 |
| Gentamicin | R | R | R | R | R | 4 |
| Imipenem | R | R | R | R | R | ≥ 16 |
| Meropenem | R | R | R | R | R | ≥ 16 |
| Piperacillin | R | R | R | R | R | ≥ 128 |

For the *e*-AST, BMD and VITEK 2 test: S, susceptible; I, intermediate; R, resistant.

| ***Pseudomonas aeruginosa* (PA1)** | | | | | | |
| --- | --- | --- | --- | --- | --- | --- |
| Antibiotic (μg/ml) | *e*-AST | | | BMD | Vitek | MIC (μg/ml) |
|  | Test 1 | Test 2 | Test 3 |  |  |  |
| Amikacin | S | S | S | S | R | ≥ 64 |
| Ampicillin | R | R | R | R | - | - |
| Aztreonam | R | R | R | R | I | 16 |
| Cefepime | R | R | R | R | R | ≥ 64 |
| Cefotaxime | R | R | R | R | - | - |
| Ceftazidime | R | R | R | R | R | ≥ 64 |
| Ciprofloxacin | R | R | R | R | R | ≥ 4 |
| Gentamicin | R | R | R | R | R | ≥ 16 |
| Imipenem | R | R | R | R | R | ≥ 16 |
| Meropenem | R | R | R | R | R | ≥ 16 |
| Piperacillin | R | R | R | R | R | ≥ 128 |

For the *e*-AST, BMD and VITEK 2 test: S, susceptible; I, intermediate; R, resistant.

| ***Pseudomonas aeruginosa* (PA2)** | | | | | | | | |
| --- | --- | --- | --- | --- | --- | --- | --- | --- |
| Antibiotic (μg/ml) | *e*-AST | | | | | BMD | Vitek | MIC (μg/ml) |
|  | Test 1 | Test 2 | Test 3 | Test 4 | Test 5 |  |  |  |
| Amikacin | S | S | S | S | S | S | S | ≤ 2 |
| Ampicillin | R | R | R | R | R | R | - | - |
| Aztreonam | R | R | R | R | R | R | R | ≥ 64 |
| Cefepime | I | R | R | R | R | R | I | 8 |
| Cefotaxime | R | R | R | R | R | R | - | - |
| Ceftazidime | R | R | R | R | R | R | I | 16 |
| Ciprofloxacin | R | R | R | R | R | R | R | ≥ 4 |
| Gentamicin | S | S | S | S | S | S | S | 2 |
| Imipenem | R | R | R | R | R | R | S | 1 |
| Meropenem | R | R | R | I | R | R | R | ≤ 0.25 |
| Piperacillin | R | R | R | R | I | R | I | ≥ 64 |

For the *e*-AST, BMD and VITEK 2 test: S, susceptible; I, intermediate; R, resistant.

| ***Pseudomonas aeruginosa* (PA3)** | | | | | | |
| --- | --- | --- | --- | --- | --- | --- |
| Antibiotic (μg/ml) | *e*-AST | | | BMD | Vitek | MIC (μg/ml) |
|  | Test 1 | Test 2 | Test 3 |  |  |  |
| Amikacin | S | S | S | S | S | ≤ 2 |
| Ampicillin | R | R | R | R | - | - |
| Aztreonam | R | R | R | R | I | 16 |
| Cefepime | S | S | S | S | S | 4 |
| Cefotaxime | R | R | R | R | - | - |
| Ceftazidime | R | S | S | S | S | 8 |
| Ciprofloxacin | S | S | S | S | S | ≤ 0.25 |
| Gentamicin | S | S | S | S | S | ≤ 1 |
| Imipenem | S | S | S | S | S | ≥ 2 |
| Meropenem | S | S | S | S | S | ≤ 0.25 |
| Piperacillin | R | R | R | R | R | ≥ 128 |

For the *e*-AST, BMD and VITEK 2 test: S, susceptible; I, intermediate; R, resistant.

| ***Pseudomonas aeruginosa* (PA4)** | | | | | | |
| --- | --- | --- | --- | --- | --- | --- |
| Antibiotic (μg/ml) | *e*-AST | | | BMD | Vitek | MIC (μg/ml) |
|  | Test 1 | Test 2 | Test 3 |  |  |  |
| Amikacin | S | S | S | S | S | ≤ 2 |
| Ampicillin | R | R | R | R | - | - |
| Aztreonam | R | R | R | R | R | 32 |
| Cefepime | R | R | R | R | I | 8 |
| Cefotaxime | R | R | R | R | - | - |
| Ceftazidime | R | R | R | R | I | 16 |
| Ciprofloxacin | R | R | R | R | R | ≥ 4 |
| Gentamicin | S | S | S | S | S | 2 |
| Imipenem | R | R | R | R | R | ≤ 16 |
| Meroponem | R | R | R | R | R | ≤ 16 |
| Piperacillin | R | R | R | R | R | ≥ 128 |

For the *e*-AST, BMD and VITEK 2 test: S, susceptible; I, intermediate; R, resistant.

| ***Pseudomonas aeruginosa* (PA5)** | | | | | | |
| --- | --- | --- | --- | --- | --- | --- |
| Antibiotic (μg/ml) | *e*-AST | | | BMD | Vitek | MIC (μg/ml) |
|  | Test 1 | Test 2 | Test 3 |  |  |  |
| Amikacin | S | S | S | S | R | ≥ 4 |
| Ampicillin | R | R | R | R | - | - |
| Aztreonam | R | R | R | R | I | 16 |
| Cefepime | R | R | R | R | R | ≥ 64 |
| Cefotaxime | R | R | R | R | - | - |
| Ceftazidime | R | R | R | R | R | ≥ 64 |
| Ciprofloxacin | R | R | R | R | R | ≥ 4 |
| Gentamicin | R | R | R | R | R | ≤ 16 |
| Imipenem | R | S | I | R | R | ≤ 16 |
| Meroponem | R | R | R | R | R | ≤ 16 |
| Piperacillin | R | R | R | R | R | ≥ 128 |

For the *e*-AST, BMD and VITEK 2 test: S, susceptible; I, intermediate; R, resistant.

| ***Klebpsiella pneumoniae* (KPN1) - ESBL** | | | | | | |
| --- | --- | --- | --- | --- | --- | --- |
| Antibiotic (μg/ml) | *e*-AST | | | BMD | Vitek | MIC (μg/ml) |
|  | Test 1 | Test 2 | Test 3 |  |  |  |
| Amikacin | R | R | R | R | R | ≥64 |
| Ampicillin | R | R | R | R | R | ≥32 |
| Aztreonam | R | R | R | R | R | ≥32 |
| Cefepime | R | R | R | R | R | 4 |
| Cefotaxime | R | R | R | R | R | ≥64 |
| Ceftazidime | R | R | R | R | R | ≥64 |
| Ciprofloxacin | R | R | R | R | R | ≥4 |
| Gentamicin | R | R | R | R | R | ≤16 |
| Imipenem | S | S | S | S | S | ≤0.25 |
| Meropenem | S | I | S | S | - | - |
| Piperacillin | R | R | R | R | - | - |

For the *e*-AST, BMD and VITEK 2 test: S, susceptible; I, intermediate; R, resistant.

| ***Klebpsiella pneumoniae* (KPN2) - ESBL** | | | | | | |
| --- | --- | --- | --- | --- | --- | --- |
| Antibiotic (μg/ml) | *e*-AST | | | BMD | Vitek | MIC (μg/ml) |
|  | Test 1 | Test 2 | Test 3 |  |  |  |
| Amikacin | S | S | S | S | S | ≤ 2 |
| Ampicillin | R | R | R | R | R | ≥ 32 |
| Aztreonam | R | R | R | R | R | ≥ 64 |
| Cefepime | R | R | R | R | R | ≥ 64 |
| Cefotaxime | R | R | R | R | R | ≥ 64 |
| Ceftazidime | R | R | R | R | R | 16 |
| Ciprofloxacin | R | R | R | R | R | ≥ 4 |
| Gentamicin | R | R | R | R | R | ≤ 16 |
| Imipenem | I | I | I | I | S | ≤ 0.25 |
| Meropenem | I | I | S | I | - | - |
| Piperacillin | R | R | R | R | - | - |

For the *e*-AST, BMD and VITEK 2 test: S, susceptible; I, intermediate; R, resistant.

| ***Klebpsiella pneumoniae* (KPN3)** | | | | | | |
| --- | --- | --- | --- | --- | --- | --- |
| Antibiotic (μg/ml) | *e*-AST | | | BMD | Vitek | MIC (μg/ml) |
|  | Test 1 | Test 2 | Test 3 |  |  |  |
| Amikacin | S | S | S | S | S | ≤ 2 |
| Ampicillin | R | R | R | R | R | ≥ 32 |
| Aztreonam | S | S | S | S | S | ≤ 1 |
| Cefepime | S | S | S | S | S | ≤ 1 |
| Cefotaxime | S | S | S | S | S | ≤ 1 |
| Ceftazidime | S | S | S | S | S | ≤ 1 |
| Ciprofloxacin | S | S | S | S | S | ≤ 0.25 |
| Gentamicin | S | S | S | S | S | ≤ 1 |
| Imipenem | S | S | S | S | S | ≤ 0.25 |
| Meropenem | S | S | S | S | - | - |
| Piperacillin | R | R | R | R | - | - |

For the *e*-AST, BMD and VITEK 2 test: S, susceptible; I, intermediate; R, resistant.

| ***Klebpsiella pneumoniae* (KPN4) - ESBL** | | | | | | |
| --- | --- | --- | --- | --- | --- | --- |
| Antibiotic (μg/ml) | *e*-AST | | | BMD | Vitek | MIC (μg/ml) |
|  | Test 1 | Test 2 | Test 3 |  |  |  |
| Amikacin | S | S | S | S | S | ≤ 2 |
| Ampicillin | R | R | R | R | R | ≥ 32 |
| Aztreonam | R | R | R | R | R | ≥ 64 |
| Cefepime | S | S | S | S | S | ≤ 1 |
| Cefotaxime | R | R | R | R | R | 8 |
| Ceftazidime | R | R | R | R | R | ≥ 64 |
| Ciprofloxacin | R | R | R | R | R | ≥ 4 |
| Gentamicin | R | I | I | I | S | ≤ 1 |
| Imipenem | R | R | R | R | S | ≤ 0.25 |
| Meropenem | I | S | S | S | - | - |
| Piperacillin | R | R | R | R | - | - |

For the *e*-AST, BMD and VITEK 2 test: S, susceptible; I, intermediate; R, resistant.

| ***Klebpsiella pneumoniae* (KPN 5) - ESBL** | | | | | | | |
| --- | --- | --- | --- | --- | --- | --- | --- |
| Antibiotic (μg/ml) | *e*-AST | | | | BMD | Vitek | MIC (μg/ml) |
|  | Test 1 | Test 2 | Test 3 | Test 4 |  |  |  |
| Amikacin | S | S | S | S | S | S | ≤2 |
| Ampicillin | R | R | R | R | R | R | ≥32 |
| Aztreonam | R | R | R | R | R | R | ≥64 |
| Cefepime | R | R | R | R | R | R | ≥64 |
| Cefotaxime | R | R | R | R | R | R | ≥64 |
| Ceftazidime | R | R | R | R | R | R | ≥64 |
| Ciprofloxacin | R | R | R | R | R | R | ≥4 |
| Gentamicin | S | S | I | S | S | S | ≤1 |
| Imipenem | S | S | S | S | S | S | ≤0.25 |
| Meropenem | S | S | S | S | S | - | - |
| Piperacillin | R | R | R | R | R | - | - |

For the *e*-AST, BMD and VITEK 2 test: S, susceptible; I, intermediate; R, resistant.

| ***S. aureus* (C970)** | | | | | | |
| --- | --- | --- | --- | --- | --- | --- |
| Antibiotic (μg/ml) | *e*-AST | | | BMD | Vitek | MIC (μg/ml) |
|  | Test 1 | Test 2 | Test 3 |  |  |  |
| Ampicillin | R | R | R | R | - | - |
| Ciprofloxacin | S | S | S | S | S | ≤0.5 |
| Clindamycin | S | S | S | S | S | ≤0.25 |
| Erythromycin | S | S | S | S | S | ≤0.25 |
| Gentamicin | S | S | S | S | - | - |
| Linezolid | S | S | S | S | S | 3 |
| Muprirocin | S | S | S | S | S | ≤2 |
| Oxacillin | S | S | S | S | S | 0.5 |
| Teicoplanin | S | S | S | S | S | ≤0.25 |
| Tetracycline | S | S | S | S | S | ≤1 |
| Vancomycin | S | S | S | S | S | ≤0.5 |

For the *e*-AST, BMD and VITEK 2 test: S, susceptible; I, intermediate; R, resistant.

| ***S. aureus* (R4308)** | | | | | | |
| --- | --- | --- | --- | --- | --- | --- |
| Antibiotic (μg/ml) | *e*-AST | | | BMD | Vitek | MIC (μg/ml) |
|  | Test 1 | Test 2 | Test 3 |  |  |  |
| Ampicillin | R | R | R | R | - | - |
| Ciprofloxacin | R | R | R | R | S | ≤ 1 |
| Clindamycin | S | S | S | S | S | ≤ 0.25 |
| Erythromycin | S | S | S | S | S | ≤ 0.25 |
| Gentamicin | S | S | S | S | - | - |
| Linezolid | S | S | S | S | S | 2 |
| Muprirocin | R | R | R | R | S | ≤ 2 |
| Oxacillin | R | R | R | R | S | ≤ 0.25 |
| Teicoplanin | R | R | R | R | S | ≤ 0.5 |
| Tetracycline | S | S | S | S | S | ≤ 1 |
| Vancomycin | S | S | S | S | S | 1 |

For the *e*-AST, BMD and VITEK 2 test: S, susceptible; I, intermediate; R, resistant.

| ***S. aureus* (P101)** | | | | | | |
| --- | --- | --- | --- | --- | --- | --- |
| Antibiotic (μg/ml) | *e*-AST | | | BMD | Vitek | MIC (μg/ml) |
|  | Test 1 | Test 2 | Test 3 |  |  |  |
| Ampicillin | R | R | R | R | - | - |
| Ciprofloxacin | R | R | R | R | S | ≤0.5 |
| Clindamycin | S | S | S | S | S | ≤0.25 |
| Erythromycin | S | S | S | S | S | 0.5 |
| Gentamicin | S | S | S | S | - | - |
| Linezolid | S | S | S | S | S | 2 |
| Muprirocin | R | R | R | R | S | ≤2 |
| Oxacillin | R | R | R | R | S | 0.5 |
| Teicoplanin | S | S | S | S | S | ≤0.5 |
| Tetracycline | S | S | S | S | S | ≤1 |
| Vancomycin | S | S | S | S | S | 1 |

For the *e*-AST, BMD and VITEK 2 test: S, susceptible; I, intermediate; R, resistant.

| ***S. aureus* (R4319)** | | | | | | |
| --- | --- | --- | --- | --- | --- | --- |
| Antibiotic (μg/ml) | *e*-AST | | | BMD | Vitek | MIC (μg/ml) |
|  | Test 1 | Test 2 | Test 3 |  |  |  |
| Ampicillin | R | R | R | R | - | - |
| Ciprofloxacin | R | R | R | R | R | ≥8 |
| Clindamycin | S | S | S | I | R | ≥8 |
| Erythromycin | S | S | S | S | R | ≥8 |
| Gentamicin | S | S | S | S | - | - |
| Linezolid | S | S | S | S | S | 2 |
| Muprirocin | R | R | R | R | S | 4 |
| Oxacillin | R | R | R | R | S | 32 |
| Teicoplanin | S | S | S | S | S | 2 |
| Tetracycline | R | R | R | R | R | ≥16 |
| Vancomycin | S | S | S | S | S | ≤0.5 |

For the *e*-AST, BMD and VITEK 2 test: S, susceptible; I, intermediate; R, resistant.

| ***S. aureus* (T82)** | | | | | | |
| --- | --- | --- | --- | --- | --- | --- |
| Antibiotic (μg/ml) | *e*-AST | | | BMD | Vitek | MIC (μg/ml) |
|  | Test 1 | Test 2 | Test 3 |  |  |  |
| Ampicillin | R | R | R | R | - | - |
| Ciprofloxacin | R | R | R | R | R | ≥8 |
| Clindamycin | R | R | R | R | R | ≥8 |
| Erythromycin | R | R | R | R | R | ≥8 |
| Gentamicin | R | R | R | R | - | - |
| Linezolid | S | S | S | S | S | 4 |
| Muprirocin | R | R | R | R | S | 4 |
| Oxacillin | R | R | R | R | R | ≥4 |
| Teicoplanin | S | S | S | S | S | ≤0.5 |
| Tetracycline | R | R | R | R | R | ≥16 |
| Vancomycin | S | S | S | S | S | ≤0.5 |

For the *e*-AST, BMD and VITEK 2 test: S, susceptible; I, intermediate; R, resistant.

| ***E. faecalis* (U5554)** | | | | | | |
| --- | --- | --- | --- | --- | --- | --- |
| Antibiotic (μg/ml) | *e*-AST | | | BMD | Vitek | MIC (μg/ml) |
|  | Test 1 | Test 2 | Test 3 |  |  |  |
| Ampicillin | S | S | S | S | S | ≤2 |
| Ciprofloxacin | S | S | S | S | S | 1 |
| Clindamycin | S | S | S | S | - | - |
| Erythromycin | I | I | I | I | S | 0.5 |
| Gentamicin | S | S | S | S | S | - |
| Linezolid | S | S | S | S | S | 2 |
| Muprirocin | R | R | R | R | - | - |
| Oxacillin | R | R | R | R | - | - |
| Teicoplanin | S | S | S | S | S | ≤0.5 |
| Tetracycline | S | S | S | S | S | ≥16 |
| Vancomycin | S | S | S | S | S | 1 |

For the *e*-AST, BMD and VITEK 2 test: S, susceptible; I, intermediate; R, resistant.

| ***E. faecalis* (U5179)** | | | | | | |
| --- | --- | --- | --- | --- | --- | --- |
| Antibiotic (μg/ml) | *e*-AST | | | BMD | Vitek | MIC (μg/ml) |
|  | Test 1 | Test 2 | Test 3 |  |  |  |
| Ampicillin | S | S | S | S | S | ≤2 |
| Ciprofloxacin | S | S | S | S | S | ≤0.5 |
| Clindamycin | S | S | S | S | - | - |
| Erythromycin | I | I | I | I | I | 2 |
| Gentamicin | S | S | S | S | S | - |
| Linezolid | S | S | S | S | S | 0.5 |
| Muprirocin | R | R | R | R | - | - |
| Oxacillin | R | R | R | R | - | - |
| Teicoplanin | S | S | S | S | S | ≤0.5 |
| Tetracycline | R | R | R | R | R | ≤1 |
| Vancomycin | S | S | S | S | S | 1 |

For the *e*-AST, BMD and VITEK 2 test: S, susceptible; I, intermediate; R, resistant.

| ***E. faecalis* (U5064)** | | | | | | |
| --- | --- | --- | --- | --- | --- | --- |
| Antibiotic (μg/ml) | *e*-AST | | | BMD | Vitek | MIC (μg/ml) |
|  | Test 1 | Test 2 | Test 3 |  |  |  |
| Ampicillin | S | S | S | S | S | ≤2 |
| Ciprofloxacin | S | S | S | S | R | ≥8 |
| Clindamycin | S | S | S | S | - | - |
| Erythromycin | S | I | I | S | R | ≥8 |
| Gentamicin | S | S | S | S | S | - |
| Linezolid | S | S | S | S | S | 2 |
| Muprirocin | R | R | R | R | - | - |
| Oxacillin | R | R | R | R | - | - |
| Teicoplanin | S | S | S | S | S | ≤0.5 |
| Tetracycline | I | I | I | R | R | ≥16 |
| Vancomycin | S | S | S | S | S | 1 |

For the *e*-AST, BMD and VITEK 2 test: S, susceptible; I, intermediate; R, resistant.

| ***E. faecalis* (U4879)** | | | | | | |
| --- | --- | --- | --- | --- | --- | --- |
| Antibiotic (μg/ml) | *e*-AST | | | BMD | Vitek | MIC (μg/ml) |
|  | Test 1 | Test 2 | Test 3 |  |  |  |
| Ampicillin | S | S | S | S | S | ≤2 |
| Ciprofloxacin | R | R | R | R | R | ≥8 |
| Clindamycin | S | S | S | S | - | - |
| Erythromycin | R | R | R | R | R | ≥8 |
| Gentamicin | R | R | R | R | R | - |
| Linezolid | S | S | S | S | S | 2 |
| Muprirocin | R | R | R | R | - | - |
| Oxacillin | R | R | R | R | - | - |
| Teicoplanin | S | S | S | S | S | ≤0.5 |
| Tetracycline | R | R | R | R | R | ≥16 |
| Vancomycin | S | S | S | S | S | 2 |

For the *e*-AST, BMD and VITEK 2 test: S, susceptible; I, intermediate; R, resistant.

| ***E. faecalis* (U12138)** | | | | | | |
| --- | --- | --- | --- | --- | --- | --- |
| Antibiotic (μg/ml) | *e*-AST | | | BMD | Vitek | MIC (μg/ml) |
|  | Test 1 | Test 2 | Test 3 |  |  |  |
| Ampicillin | S | S | S | S | S | ≤2 |
| Ciprofloxacin | R | R | R | R | R | 1 |
| Clindamycin | R | R | R | R | - | - |
| Erythromycin | I | I | I | S | S | ≤0.25 |
| Gentamicin | S | S | S | S | S | - |
| Linezolid | S | S | S | S | S | 2 |
| Muprirocin | R | R | R | R | - | - |
| Oxacillin | R | R | R | R | - | - |
| Teicoplanin | S | S | S | S | S | ≤0.5 |
| Tetracycline | I | R | R | R | S | ≤1 |
| Vancomycin | S | S | S | S | S | 1 |

For the *e*-AST, BMD and VITEK 2 test: S, susceptible; I, intermediate; R, resistant.
